# Leukocyte activation primes fibrinogen for proteolysis by mitochondrial oxidative stress

**DOI:** 10.1016/j.redox.2022.102263

**Published:** 2022-02-10

**Authors:** Chang Yeop Han, Trey J. Pichon, Xu Wang, Kristyn M. Ringgold, Alexander E. St John, Susan A. Stern, Nathan J. White

**Affiliations:** a Department of Emergency Medicine and Resuscitation Engineering Science Unit, Harborview Medical Center, USA; b Department of Bioengineering and Molecular Engineering and Sciences Institute, University of Washington, USA; c Bloodworks Northwest Research Institute, Seattle, WA, USA

**Keywords:** Coagulopathy, Inflammation, Fibrinogen, Reactive oxygen species, Oxidation, Antioxidants, MPO, myeloperoxidase, ROS, reactive oxygen species, NOX, NADPH oxidase, NO, nitric oxide, NOS, nitric oxide synthase, NAC, N-acetylcysteine, Mito-Tempo, 1 mmol/L, 2-(2,2,6,6-Tetramethylpiperidin-1-oxyl-4-ylamino)-2-oxoethyl)triphenylphosphonium chloride, L-NMMA, N^G^-monomethyl-l-arginine acetate, 4-ABAH, 4-aminobenzoic acid hydrazide, apocynin, 1-(4-hydroxy-3-methoxyphenyl)-ethanone, SOD, superoxide dismutase, HOCl, hypochlorous acid, i.v., intravenous, TXA, tranexamic acid, TIC, trauma-induced coagulopathy, LPS, lipopolysaccharides

## Abstract

Critical illness leads to rapid fibrinogen consumption, hyperfibrinolysis, and coagulopathy that exacerbates bleeding and increases mortality. Immune cell activation and inflammation are associated with coagulopathy after injury but play an undetermined role. We performed high dimensional immunophenotyping and single-cell imaging flow cytometry to investigate for a pathophysiological mechanism governing the effects of leukocyte-associated inflammation on fibrinogen function. Fibrinogen was oxidized early, followed by its degradation after 3 hours of lipopolysaccharides (LPS)-induced sterile inflammation in a rat model *in vivo*. Fibrinogen incubated with human leukocytes activated by TNFα was similarly oxidized, and later proteolyzed after 3 hours *in vitro*. TNFα induced mitochondrial superoxide generation from neutrophils and monocytes, myeloperoxidase (MPO)-derived reactive oxygen species (ROS) from neutrophils, and nitric oxide from lymphocytes and monocytes. Inhibition of mitochondrial superoxide prevented oxidative modification and proteolysis of fibrinogen, whereas inhibition of MPO attenuated only fibrinogen proteolysis. Quenching of both mitochondrial superoxide and MPO-derived ROS prevented coagulopathy better than tranexamic acid. Collectively, these findings indicate that neutrophil and monocyte mitochondrial superoxide generation can rapidly oxidize fibrinogen as a priming step for fibrinogen proteolysis and coagulopathy during inflammation.

## Highlights

• Leukocyte-induced inflammation first oxidizes, then degrades fibrinogen.
• Fibrinogen is primed for proteolysis by a process that involves mitochondrial and myeloperoxidase-derived oxidative stress.
• Inhibition of oxidative stress by antioxidants can prevent both fibrinogen oxidation and degradation.

## Graphical abstract

**Figure:**
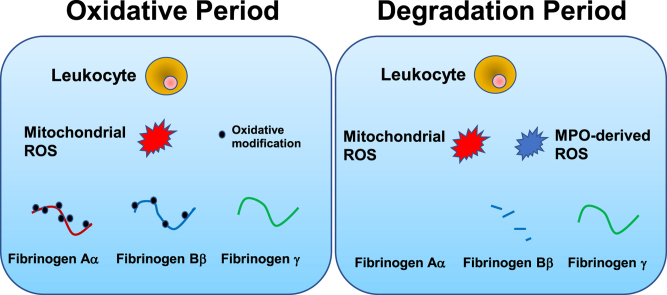

## 1 Introduction

Coagulopathy is a bleeding disorder that impairs clot formation, causing excessive bleeding and leading to life-threatening events [1,2]. Several factors contribute to this hypocoagulability, including coagulation factor inhibition, platelet dysfunction, fibrinogen consumption, and hyperfibrino(geno)lysis [[3], [4], [5], [6]]. Among those causes, inflammation through endothelial and immune system activation is presumed to be a key initiator that contributes to the underlying mechanism of both sepsis-induced and trauma-induced coagulopathy (TIC) [[5], [6], [7], [8], [9], [10]].

Fibrinogen is a glycoprotein complex, made by the liver, that normally circulates in the range of 2–4 mg/mL in blood [11,12]. It is composed of two trimers (Aα, Bβ and γ) and converted enzymatically by thrombin to fibrin, forming the structural basis of blood clots [[13], [14], [15]]. Depletion and degradation of fibrinogen is strongly associated with coagulopathy, morbidity, and mortality after severe injury and infection [1]. We previously demonstrated that oxidative modification of fibrinogen directly impairs fibrin clot formation *in vitro* and that oxidative modifications are increased in trauma patients with TIC [16,17]. This suggests that oxidative stress may play a role in fibrinogen function during acute inflammatory states. However, there is little or no information available regarding the potential mechanisms involved.

Leukocytes, consisting of monocytes, lymphocytes and neutrophils, can influence blood coagulation and fibrinolysis in many ways. Leukocytes can activate thrombosis by releasing tissue factor [18,19], and by activating platelets and endothelial cells [20,21]. They can also influence fibrinolysis by expressing urokinase-type plasminogen activator (uPA) and elastase [[22], [23], [24]], thus contributing to either hypercoagulability or hypocoagulability depending on the situation [25]. Leukocytes can also create oxidative stress by generation of reactive oxygen species (ROS). ROS can be generated in many forms depending on the source, for example, superoxide from mitochondria and NADPH oxidase (NOX), nitric oxide (NO) from nitric oxide synthase (NOS) and hypochlorous acid (HOCl) from myeloperoxidase (MPO). These oxidants can both modify blood proteins directly and regulate inflammatory signaling pathways.

In this study, we investigated the direct effects of leukocyte-derived inflammation and ROS on fibrinogen form and function. We first demonstrated that fibrinogen oxidation, proteolysis, and coagulopathy were present and associated with increased TNFα levels in a rat model of sterile inflammation induced by lipopolysaccharides (LPS) infusion. To further investigate potential mechanisms, we then exposed human fibrinogen to human leukocytes activated by TNFα *in vitro* and used single cell flow cytometry to measure ROS generation. We found that ROS were primarily generated by neutrophils and monocytes, sequentially generating mitochondrial superoxide, followed by MPO-derived ROS, and NO/NOX-derived ROS. The ROS generated could oxidize fibrinogen, were associated with its proteolytic degradation, and could induce coagulopathy. Mitochondrial superoxide and MPO-derived ROS also mediated NFκB *trans*-activation and leukocyte TNFα secretion. Only antioxidants inhibiting mitochondrial superoxide and MPO could block fibrinogen oxidation and prevent its degradation. Collectively, these data suggest that mitochondrial superoxide and MPO-derived ROS from neutrophils and monocytes can directly oxidatively modify fibrinogen, priming it for enzymatic degradation during inflammation.

## 2 Material and methods

### 2.1 Animals

To investigate the alteration of fibrinogen and hemostasis *in vivo* during early inflammation, male Sprague-Dawley rats weighing about 300 g (n = 5, Charles River Laboratories) were intravenously (i.v.) injected with LPS (1.5 mg/kg, 111:B4, Sigma) for indicated time periods. All experimental procedures were undertaken with approval from the Institutional Animal Care and Use Committee of the University of Washington (Protocol #4329-05).

### 2.2 OxyBlot and western blot analysis

The extent of oxidative modification of fibrinogen was analyzed using the OxyBlot protein oxidation detection kit (Millipore) according to manufacturer's instruction. Carbonyl groups induced by oxidative modification are derivatized to 2,4-dinitrophenylhydrazone (DNP-hydrazone) by reaction with 2,4-dinitrophenylhydrazine (DNPH) and the DNP-derivatized proteins detected by western blot analysis. Briefly, isolated plasma from LPS-injected rats or culture conditioned media from human leukocytes were reduced and electrophoresed on a 4–12% gradient SDS-PAGE gel. Proteins were then transferred to nitrocellulose membranes. The membranes were incubated with a rabbit anti-DNP antibody, and then with a horseradish peroxidase-conjugated goat anti-rabbit secondary antibody.

Plasma from LPS-injected rats or culture conditioned media from human leukocytes were also analyzed by western blot using anti-fibrinogen Aα, Bβ and γ antibodies. The samples were electrophoresed on a 4–12% gradient SDS-PAGE gel and then transferred to nitrocellulose membranes using a Bio-Rad Mini-Transfer Cell. After incubation with the blocking buffer (10% Aqua Block, EastCoast Bio in PBS-T containing 0.05% Tween-20) for 2 h at room temperature, membranes were incubated with a mouse monoclonal antibody against fibrinogen Aα (R&D, 949380, 1:1000), a rabbit monoclonal antibody against fibrinogen Bβ (Abcam, EPR1814-84, 1:1000), and a rabbit monoclonal antibody against fibrinogen γ (Abcam, EPR30841, 1:1000), respectively, overnight at 4 °C in blocking buffer. PBS-T was used for all washing steps. After incubation with the horseradish peroxidase-conjugated secondary antibody for 1 h at room temperature, membranes were washed and scanned by using a GE ImageQuant 350 and software (GE). After western blot, each band corresponding to the apparent fibrinogen Aα, Bβ and γ was excised and subjected to in-blot trypsin digestion in order to confirm whether anti-DNP and anti-fibrinogen antibodies detect the exactly matched proteins. Tryptic peptides were then extracted and analyzed by matrix-assisted-laser desorption (MALDI) time-of-flight (TOF) tandem mass spectrometry as described previously [26].

### 2.3 Multiplex cytokines measurement

Cytokines and chemokines (IL1α, IL1β, IL6, IL10, IL12, IL17, IL18, IL33, GM-CSF, MCP-1, CXCL1, IFNγ, and TNFα) from LPS-injected rat plasma were measured using the multiplex immunoassay LEGENDplex rat Inflammation Panel (BioLegend) following the manufacturer's instructions. Samples were read using an LSRII (BD) flow cytometer and FlowJo. v10 (Treestar). Data analysis was performed with the LEGENDplex Data Analysis Software v.8 (BioLegend).

### 2.4 Rotational Thromboelastometry (ROTEM)

Citrated whole rat blood was recalcified to 10 mmol/L with 0.2 mol/L of calcium chloride and then transferred to 37 °C ROTEM minicups (Werfern) for assessment via ROTEM using EXTEM and FIBTEM assays as described previously [27]. EXTEM (tissue factor triggered extrinsic pathway) evaluated the combined influence of thrombin, platelets, and fibrin on clot formation, while FIBTEM, containing the platelet inhibitor, cytochalasin D, isolated the contribution of fibrin to clot formation. ROTEM was used to measure clotting time (CT), defined as the time between reagent addition to clot formation, α-angle, which reflects the rate of clot formation, maximum clot firmness (MCF), lysis index-30 min (LI-30), the percentage of MCF retained 30 min after initiation of clot formation, and maximum lysis (ML), the percentage of clot strength lost compared to the MCF at the end of analysis. The thromboelastometry assay was carried out in 60 min, and data were analyzed via ROTEM software (Werfern).

### 2.5 Human donors

To investigate whether leukocytes from human subjects affect fibrinogen and disrupt coagulation, whole blood was isolated from 6 healthy subjects (3 male, 3 female, 44.5 ± 14.18 years mean ± SD) at the Bloodworks Northwest Research Institute, Seattle, Washington. All donors provided written informed consent and authorization for blood draws and release of medical information (Protocol # BT001).

### 2.6 Flow cytometry analysis

Whole human blood was incubated in ACK RBC lysis buffer (Thermo Fisher Scientific) for 4 min at 37 °C to remove red blood cells. Cells were incubated with NIR Zombie (1:200, Biolegend) in PBS for live/dead staining for 15 min at room temperature, followed by BV421 CD45 (1:200, clone 2D1, #368522, Biolegend), APC CD14 (1:200, clone 63D3, #367118, Biolegend), BV605 CD16b (1:200, clone CLB-gran 11.5, #735143 BD), PE/Cy7 CD19 (1:200, clone HIB19, #302216, Biolegend), PE/Cy7 CD3 (1:200, clone HIT3a, #300316, Biolegend) and Fc blocker (1:100, anti-mouse CD16/32,BD) at 4 °C in the dark for 20 min, and washed in PBS/0.5% BSA. Neutrophils, lymphocytes, and monocytes were identified from peripheral blood cells of normal healthy donors with the following high dimensional immunophenotyping; Neutrophils (CD45^+^, CD3/CD19−, CD16b+, CD14^−^), lymphocytes (CD45^+^, CD3/CD19+, CD16b−, CD14^−^) and monocytes (CD45^+^, CD3/CD19−, CD16b−, CD14^−^). Analysis was performed using an LSRII (BD) flow cytometer and FlowJo. v10 (Treestar).

### 2.7 Quantification of ROS

To identify the source of ROS, isolated leukocytes (10 [6] cells) were incubated with 10 ng/mL of TNFα in RPMI 1640 media (Gibco) with or without antioxidants (N-acetylcysteine, NAC; pan antioxidant, 1 mmol/L, 2-(2,2,6,6-Tetramethylpiperidin-1-oxyl-4-ylamino)-2-oxoethyl)triphenylphosphonium chloride, Mito-Tempo; antioxidant for mitochondrial superoxide, 100 μmol/L, N^G^-Monomethyl-l-arginine acetate, L-NMMA; antioxidant for NOS, 100 μmol/L, 4-aminobenzoic acid hydrazide, 4-ABAH; antioxidant for MPO, 20 μmol/L.

1-(4-hydroxy-3-methoxyphenyl)-ethanone, apocynin; antioxidant for NOX, 10 μmol/L, all reagents from Cayman Chemical) for 3 h. Total ROS generation in TNFα-activated leukocytes was assessed as CM-H_2_DCFDA (DCF, Molecular Probes) and NO was measured using 4-amino-5-methylamino-2′,7′-difluorofluorescein (DAF-FM, Molecular Probes) as described previously [28]. Mitochondrial superoxide was measured using MitoSOX Red reagent (Molecular Probes), which is a live cell permeant that is rapidly and selectively targeted to mitochondria and, once in the mitochondria, is oxidized by superoxide and exhibits red fluorescence [28]. After washing twice in PBS, RPMI 1640 media without phenol red was added, and stained cells were analyzed by LSRII (BD) flow cytometer and FlowJo. v10 (Treestar).

### 2.8 Isolation of neutrophils, lymphocytes and monocytes

Human neutrophil-, lymphocyte-, monocyte-enriched cells were purified (>90% purity) from fresh peripheral whole blood using magnetic immunoaffinity isolation with negative-selection by antibodies conjugated to magnetic beads (MACS; Miltenyi Biotec, Germany) according to the manufacturer's instruction.

### 2.9 Measurement of MPO and NOX activity

MPO activity was measured using MPO chlorination fluorometric assay kit (Cayman Chemical) from whole lysate of leukocytes, neutrophils, lymphocytes or monocytes according to the manufacturer's instruction. MPO activity was calculated as the amount of enzyme that caused the formation of 1 pmol of fluorophore per minute at 25 °C. NOX activity was measured from membrane fraction of isolated leukocytes, neutrophils, lymphocytes or monocytes by superoxide dismutase (SOD)-inhibitable chemiluminescence as described previously [28].

### 2.10 Imaging flow cytometry analysis of NFκB *trans*-location and intracellular TNFα expression

To evaluate the extent of inflammation of TNFα-activated leukocytes treated with various antioxidants, single-cell flow imaging was performed on leukocytes stained with Alexa Fluor 488 NFκB p65 (1:200, clone K10–895.12.50, #55841, BD) and PE TNFα (1:200, clone 6401.1111, # antibodies, #340512, BD) using the Amins ImageStreamX Mark II flow cytometer (Luminex). Compensation and analysis were performed using Amnis IDEAS software 6.2 (Luminex). Focused cells were chosen based on a bright field intensity gradient of >40, followed by elimination of debris and doublets by the bright field aspect ratio vs area dot plot.

### 2.11 Thrombin and reptilase time assays

To determine whether TNFα-activated leukocytes disturb the clottability of fibrinogen, human leukocytes were incubated with 4 mg/mL of fibrinogen (Sigma) and/or various antioxidants for the indicated time points. The extent of fibrin polymerization in conditioned media from TNFα-activated leukocytes was measured as using thrombin and reptilase time using the START-4 steel ball coagulation analyzer (Diagnostica Stago, Asnières, France) as described previously [17]. Prolongation of both thrombin and reptilase times indicates the presence of altered fibrin polymerization (dysfibrinogenemia) rather than inhibition by the presence of heparins or other inhibitors affecting thrombin function.

### 2.12 Statistics

Statistical testing was chosen based upon data distribution as appropriate. Statistical tests were performed with SPSS (Windows version 19) or OriginPro software (version 8.6; Origin Laboratory). All data are shown as means ± SEM. One-way ANOVA (ANOVA) was used to compare differences between groups with Bonferroni post-hoc adjustment for multiple comparisons. Pearson coefficient was used for linear regression analysis. An overall p value < 0.05 was considered statistically significant.

## 3 Results

### 3.1 Fibrinogen is oxidized and degraded *in vivo* and coagulopathy is associated with TNFα in a rat model of sterile inflammation

**Fig. 1:**
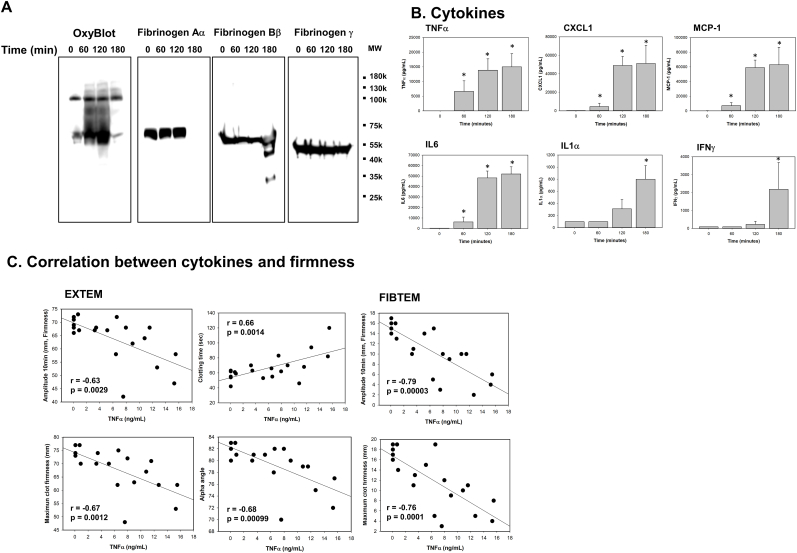
**Fibrinogen is oxidized and degraded in endotoxin-induced inflammation, causing coagulopathy in rats.** Whole blood and plasma were collected at the indicated time points from LPS-injected male rats (n = 5). (*A*) Plasma was analyzed by OxyBlot and immunoblotting using fibrinogen Aα, Bβ and γ – specific antibodies. Data are representative of 3 independent experiments. (*B*) Cytokines were measured from plasma at the indicated time points by multiplex cytokine analysis kit (n = 5, mean ± standard error of the mean (SEM)). *p < 0.05 vs. control. ANOVA and Bonferroni post-hoc test. (*C*) Amplitude, clotting time, α-angle, and MCF were measured in FIBTEM and EXTEM. Relationships between TNFα and amplitude, clotting time, α-angle, or MCF, were determined by linear regression analysis using Pearson correlation coefficient.

To determine if fibrinogen is directly affected by inflammation *in vivo*, we induced sterile inflammation by injecting endotoxin i.v. into rats, and sequentially measured cytokines and fibrinogen oxidative modification and degradation. We found that fibrinogen was rapidly oxidized as measured by carbonyl band-staining using OxyBlot (Fig. 1A). Fibrinogen Aα was oxidized first within 1-h, followed by fibrinogen Bβ at 2-h. Fibrinogen γ was resistant to oxidation at all time points (Fig. 1A). Fibrinogen Aα chain then became undetectable in western blotting and fibrinogen Bβ was partially degraded and fragmentized at 3-h, whereas fibrinogen γ resisted proteolysis (Fig. 1A). Given these results, we arbitrarily assigned two time periods. The first period was characterized by extensive oxidation without degradation before 3 h (oxidative period). The second period was characterized by extensive fibrinogen fragmentation and degradation after 3 h (degradation period).

Many cytokines are involved in both hypercoagulability and hypocoagulability [8,10,25,29]. To identify specific cytokines associated with fibrinogen function during inflammation, we concurrently measured a 13-cytokine panel in plasma (Fig. 1B). Among 13 pro-inflammatory cytokines measured (IL1α, IL1β, IL6, IL10, IL12, IL17, IL18, IL33, GM-CSF, MCP-1, CXCL1, IFNγ, and TNFα), only 6 cytokines and chemokines (IFNγ, CXCL1, MCP1, IL1α, IL6 and TNFα) were significantly increased (Fig. 1B). TNFα was the first to increase significantly, followed by IL6 during the oxidative period. IFNγ, CXCL1,MCP-1 and IL1α became increased later during the degradation period. Significant associations were found between pro-inflammatory cytokines TNFα and IL6 and impaired clot formation measured using the ROTEM assays measured between 1 and 3 h that were present in both the EXTEM whole blood and FIBTEM fibrin-specific assays (Fig. 1C and Supplemental Fig. I). These results indicate that increased inflammation was associated with impaired clot formation and impaired fibrin polymerization specifically. Overall, these data suggest that LPS challenge *in vivo* induces early fibrinogen oxidation and later fibrinogen degradation that is mediated by increased TNFα.

### 3.2 Fibrinogen is oxidized and degraded by TNFα-activated human leukocytes *in vitro*

**Fig. 2:**
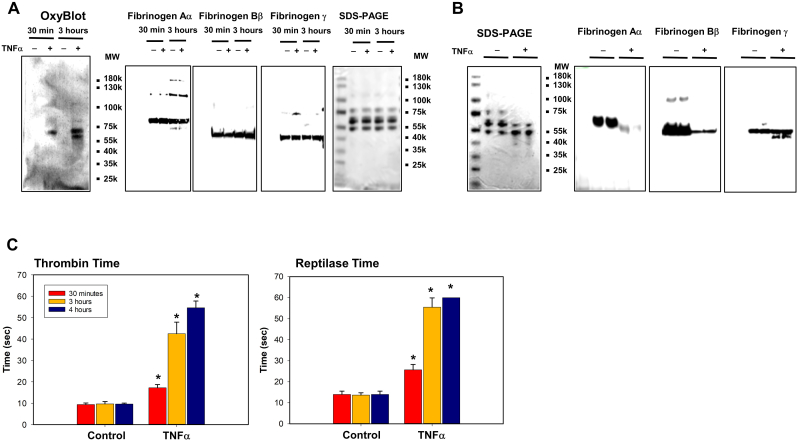
**Incubation of fibrinogen with TNFα-activated leukocytes induces oxidation followed by proteolysis of fibrinogen Αα and Ββ chains and increased thrombin and reptilase time.** TNFα-activated leukocytes from healthy donors (n = 6) were incubated with fibrinogen (4 mg/mL) during the indicated time periods. (*A* and *B*) Conditioned media at indicated time points (*A*) or 4 h (*B*) was analyzed by OxyBlot, SDS-PAGE gel and immunoblotting using fibrinogen Aα, Bβ and γ – specific antibodies. Images are representative of 3 independent experiments. (C) Thrombin and reptilase time were measured as described in methods. *p < 0.001 vs. control, ANOVA and Bonferroni post-hoc test.

To identify potential pathophysiological mechanisms involved in fibrinogen oxidation and proteolysis during inflammation, we incubated human leukocytes stimulated by TNFα with human fibrinogen *in vitro*. We chose to activate leukocytes using TNFα because it was the first pro-inflammatory cytokine to increase significantly *in vivo,* and it correlated with clot formation parameters (Fig. 1). Consistent with *in vivo* rat experiments, fibrinogen Aα-chain became oxidized within 30 min, and fibrinogen Bβ-chain was oxidized by 3 h (Fig. 2A). Fibrinogen γ-chain again remained resistant to oxidation during the oxidative period and no fibrinogen proteolysis was detected (Fig. 2A). However, by the degradation period (>3 h), fibrinogen Aα-chain was completely degraded, fibrinogen Bβ-chain, was partially degraded, and fibrinogen γ-chain remained intact at 4 h (Fig. 2B). These changes also corresponded to increased thrombin and reptilase time starting at 30 min of incubation time and substantially more so at 3 h (Fig. 2C). These data suggest that our *in vitro* model reproduced key changes in fibrinogen form and function noted in the *in vivo* sterile inflammation model.

### 3.3 Mitochondrial superoxide and MPO-related ROS are generated primarily by activated neutrophils and monocytes, followed by NO-derived ROS in all cell types

**Fig. 3:**
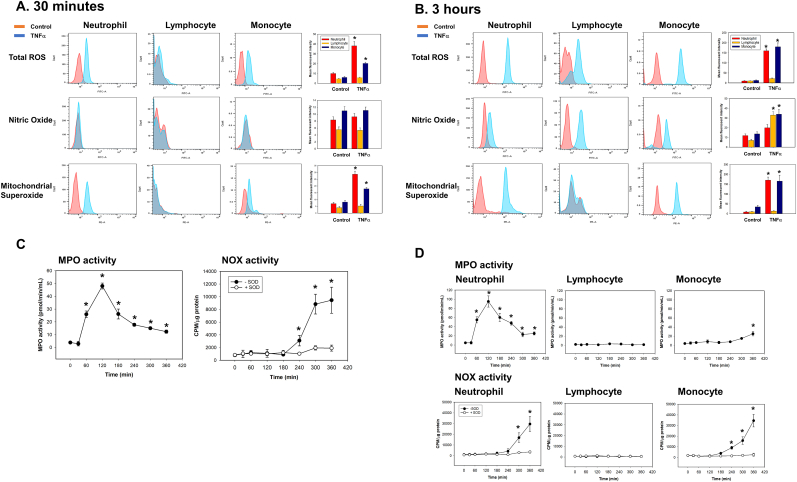
**Mitochondrial superoxide and MPO-derived ROS from neutrophils and monocytes are generated on the early onset, whereas NO and NOX-derived ROS from neutrophils, lymphocytes and monocytes are generated on the later in TNFα-activated leukocytes.** Human leukocytes from healthy donors (n = 6) were activated by TNFα (10 ng/mL) and incubated for the indicated time periods. (*A* and *B*) Total ROS, NO and mitochondrial superoxide were measured as described in methods. Each panel are representative of 6 independent experiments. Results in right panel of each row are plotted as the mean fluorescent intensity of each sample on the vertical axis with mean ± SEM. *p < 0.001 vs. control, ANOVA and Bonferroni post-hoc test. (*C* and *D*) MPO and NOX activities from leukocytes (*C*) or isolated neutrophils, lymphocytes and monocyte (*D*) were measured as described in methods (n = 6, mean ± SEM). *p < 0.001 vs. control, ANOVA and Bonferroni post-hoc test.

To identify the source of ROS in TNFα-activated human leukocytes, we identified neutrophils, lymphocytes, and monocytes by high-dimensional immunophenotyping as described in Methods (Supplemental Fig. II). In addition, to determine the molecular source of ROS generation, we specified ROS using fluorescence-based assays, measuring MPO and NOX activity in purified neutrophils, lymphocytes and monocytes as described in Methods. During the oxidative period, total ROS were generated primarily from mitochondrial superoxide by neutrophils and monocytes (Fig. 3A). During the degradation period, total ROS were increased in all cell types, but to a greater extent in neutrophils and monocytes compared to lymphocytes (Fig. 3B). Lymphocytes only generated NO as a molecular source of ROS generation (Fig. 3B). MPO-associated ROS activity was increased at 1 h and peaked at 2 h, while NOX activity was significantly increased only after 4 h (Fig. 3C). MPO activity was primarily increased in neutrophils during the oxidative period, and NOX activity was increased in both lymphocytes and monocytes during the degradation period (Fig. 3D). These data indicate that there is sequential ROS generation in TNFα-activated leukocytes. Mitochondrial superoxide is generated first in neutrophils and monocytes, later followed by MPO and NO-derived ROS in all cell types.

### 3.4 Mitochondrial superoxide from neutrophils and monocytes, and MPO-derived ROS from neutrophils contribute to total oxidative stress in TNFα-activated leukocytes

**Fig. 4:**
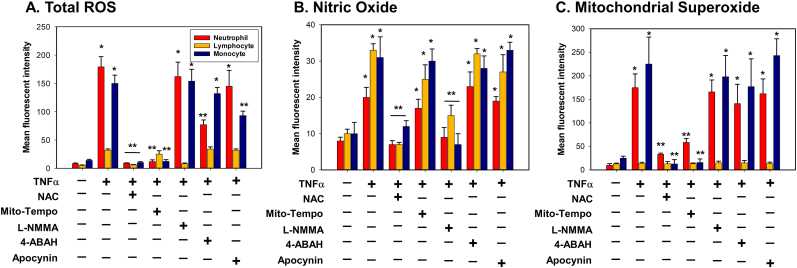
**Oxidative stress from TNFα-activated leukocytes is caused by mitochondrial superoxide and MPO-derived ROS from neutrophils and monocytes.** TNFα-activated leukocytes from healthy donors (n = 6) were incubated with indicated various antioxidants (NAC; a pan antioxidant, Mito-Tempo; an antioxidant for mitochondrial superoxide, L-NMMA; an antioxidant for NOS, 4-ABAH; an antioxidant for MPO, apocynin; an antioxidant for NOX) for 3 h. (*A-C*) Total ROS, NO and mitochondrial superoxide were measured as described in methods. Results are plotted as the mean fluorescent intensity of each sample on the vertical axis with mean ± SEM. *p < 0.001 vs. control. **p < 0.001 vs. TNFα. ANOVA and Bonferroni post-hoc test.

Given the multiple sources of ROS generated by activated leukocytes, we sought to identify the primary sources of overall oxidative stress using selective inhibition of ROS by antioxidants. In neutrophils, total ROS generation was completely inhibited by both the pan-antioxidant, NAC, and an antioxidant specific to mitochondrial superoxide, Mito-Tempo, while partially suppressed by an antioxidant to MPO-derived ROS, 4-ABAH (Fig. 4A and Supplemental Fig. III). In lymphocytes, total ROS was blocked only by a specific antioxidant to NOS, L-NMMA (Fig. 4A and Supplemental Fig. III). In monocytes, total ROS was inhibited by NAC and Mito-Tempo, but not by an antioxidant to MPO-derived ROS (Fig. 4A and Supplemental Fig. III). Antioxidants against NOX-derived ROS showed no inhibition of ROS generation in all leukocyte cell types (Fig. 4A and Supplemental Fig. III). Inhibition of NO by L-NMMA had minimal effects on overall ROS generation for all cell types. (Fig. 4B and Supplemental Fig. III). These results suggest that molecular sources of ROS generation that influence total oxidative stress in leukocytes are mitochondrial superoxide and MPO-derived ROS generated primarily by neutrophils and monocytes.

### 3.5 Mitochondrial superoxide and MPO-derived ROS from neutrophils and monocytes regulate intracellular signaling pathways in TNFα-activated leukocytes

**Fig. 5:**
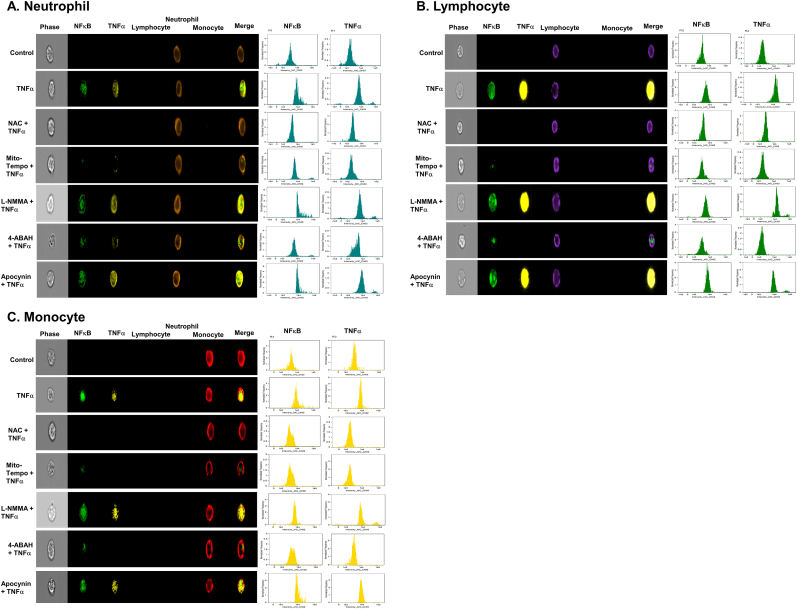
**NAC, Mito-Temp and 4-ABAH reduce inflammation (NFκB translocation and TNFα production) in TNFα-activated leukocytes.** TNFα-activated leukocytes from healthy donors (n = 6) were incubated with indicated various antioxidants as described in Fig. 4 for 3 h. (*A-C*) NFκB translocation and intracellular TNFα production was visualized and analyzed using a single-cell imaging flow cytometry in neutrophils (*A*), lymphocytes (*B*) and monocytes (*C*). Quantitative histograms are shown in right panel of each row.

The witnessed 3-h delay of fibrinogen degradation suggested that secondary intracellular activation of additional proinflammatory genes may be required to induce significant fibrinogen degradation. To examine for secondary inflammatory gene activation in leukocytes coinciding with fibrinogen degradation, we measured nuclear NFκB *trans*-location and resulting intracellular TNFα expression. NFκB is a well-studied and abundant nuclear transcription factor that governs rapid and robust increase of pro-inflammatory cytokines in leukocytes [30]. In addition, through this NFκB pathway, proteolytic enzymes could be increased. Neutrophils were stained with a CD16b antibody (brown color, 5th column, Fig. 5A), lymphocytes were stained with a CD3/19 antibody (purple color, 4th column, Fig. 5B), and monocytes were stained using a CD14 antibody (red color, 6th column, Fig. 5C). NFκB *trans*-location into the nucleus was detected using a NFκB p65 antibody (green color, 2nd column, Fig. 5A-C). Intracellular TNFα expression was detected using a TNFα antibody (yellow color, 3rd column, Fig. 5A-C). Signal transduction and pro-inflammatory gene expression judged by NFκB translocation and TNFα expression were increased in all cell types after exogenous TNFα stimulation and were inhibited by NAC, Mito-Tempo, and 4-ABAH (3rd, 4th, and 6th rows, Fig. 5A-C), but were unaffected by L-NMMA or apocynin (5th and 7th rows, Fig. 5A-C). These data suggest that mitochondrial superoxide and MPO-derived ROS triggers intracellular signal transduction and pro-inflammatory gene expression in all leukocytes.

### 3.6 Mitochondrial superoxide from neutrophils contributes to oxidation and proteolysis of fibrinogen while MPO-derived ROS contributes only to proteolysis

**Fig. 6:**
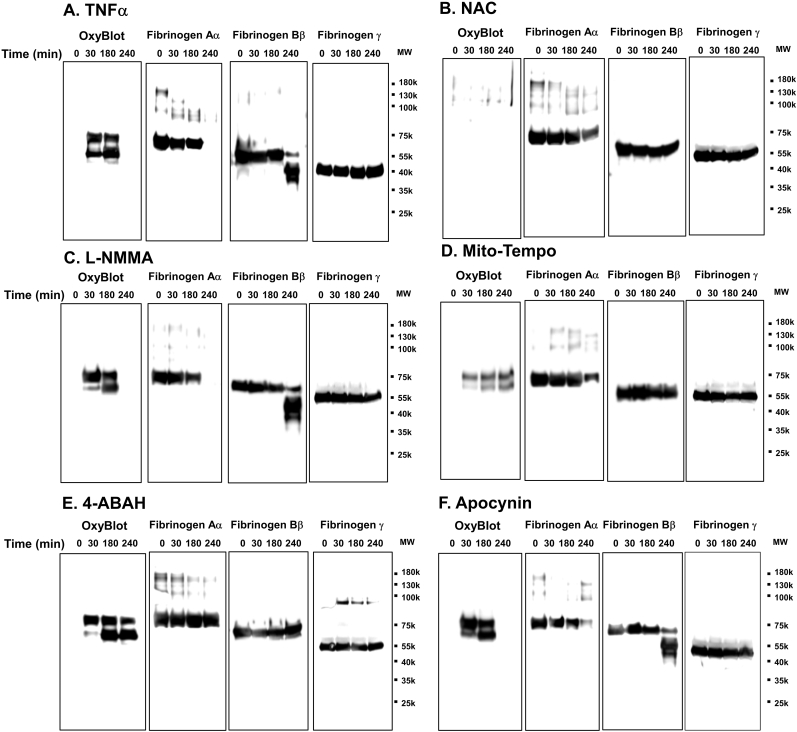
**NAC, Mito-Tempo and 4-ABAH-inhibitable ROS are responsible for oxidation and proteolysis of fibrinogen in TNFα-activated leukocytes.** TNFα-activated leukocytes from healthy donors (n = 6) were incubated with indicated various antioxidants as described in Fig. 4 for indicated time periods. (*A-F*) Conditioned media was analyzed by OxyBlot and immunoblotting using fibrinogen Aα, Bβ and γ – specific antibodies. Data images are representative of 3 independent experiments.

**Fig. 7:**
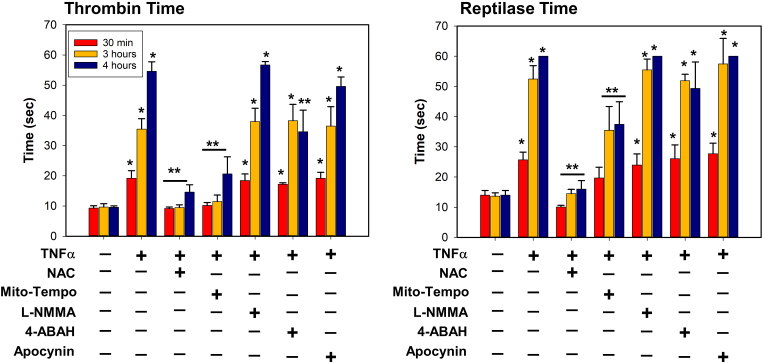
**NAC and Mito-Tempo alleviate the delay of thrombin and reptilase time from conditioned media at 30 min, 3 and 4 h, but 4-ABAH reduces thrombin and reptilase time at only 4 h in TNFα-activated leukocytes.** TNFα-activated leukocytes from healthy donors (n = 6) were incubated with fibrinogen and indicated various antioxidants as described in Fig. 4 for indicated time periods. Conditioned media was analyzed for thrombin and reptilase time were measured as described in methods (n = 6, mean ± SEM). *p < 0.001 vs. control. **p < 0.001 vs. TNFα. ANOVA and Bonferroni post-hoc test.

We then asked what types of ROS generated during leukocyte activation contributed to fibrinogen oxidation, proteolysis, and coagulopathy using targeted inhibition by antioxidants. Using OxyBlot and western blot from conditioned media treated with TNFα as a positive control (Fig. 6A), we found that; NAC completely blocked fibrinogen oxidation and degradation; Mito-Tempo decreased both fibrinogen oxidation and degradation; and the MPO-targeted antioxidant 4-ABAH, did not affect fibrinogen oxidation, but inhibited fibrinogen degradation (Fig. 6). Neither L-NMMA nor apocynin, affected fibrinogen oxidation or degradation (Fig. 6C and F). Changes in thrombin and reptilase time assays corresponded to changes seen on western blot, whereas NAC and Mito-Tempo preserved thrombin and reptilase times during the oxidative and degradation periods, and 4-ABAH only preserved thrombin and reptilase times during the later degradation period (Fig. 7). Collectively, these results suggest that mitochondrial superoxide and MPO-derived ROS can induce both direct oxidation of fibrinogen, and its subsequent degradation.

### 3.7 Inhibition of proteolysis by the lysine analog tranexamic acid (TXA) does not inhibit fibrinogen oxidation and incompletely inhibits proteolysis

To further examine the relationship between oxidation and proteolysis, we next examined the effects of tranexamic acid (TXA) on fibrinogen oxidation and proteolysis *in vitro* using the human leukocyte system. TXA is a synthetic lysine derivative that interferes with plasminogen activation by binding its lysine binding sites, reducing localization of plasminogen to fibrinogen and fibrin and plasmin activation. In large clinical trials, TXA provides a mortality benefit for bleeding trauma patients when given within 3 h of injury, but a potential net harm when given after 3 h [31]. TXA did not affect fibrinogen oxidation by OxyBlot during the oxidative period (Supplemental Fig. IV). TXA did inhibit degradation of fibrinogen Bβ-chain but did not affect degradation of fibrinogen Aα chain after 3 h (Supplemental Fig. IV). Consistent with these results, there were no effects of TXA on thrombin and reptilase times which increased significantly during the oxidative period and only partially attenuated thrombin time later during the degradation phase after 4 h (Supplemental Fig. IV). These results suggest that proteolytic enzymes inhibited by lysine analogues dot not affect early oxidation of fibrinogen by leukocytes. TXA also appears to be only partially effective at inhibiting fibrinogen proteolysis occurring after 3 h, coinciding with its therapeutic window noted in clinical trials, and suggesting that oxidation should be addressed in addition to degradation in order to maintain fibrinogen function during inflammation.

## 4 Discussion

We found that fibrinogen is directly affected by leukocyte-associated inflammation in two ways. Fibrinogen is first oxidized, then becomes proteolyzed. These events were present in a rat model of sterile inflammation *in vivo,* with oxidation occurring before, and proteolysis occurring after 3 h of LPS exposure. Further *in vitro* investigations revealed that fibrinogen oxidation was induced primarily by mitochondrial superoxide generated from neutrophils and monocytes and was confined to its Aα and Bβ chains, as was later proteolysis. We found that NO generated from lymphocytes had little or no effect on oxidation of fibrinogen. Furthermore, mitochondrial superoxide and MPO-derived oxidants played an important role in priming fibrinogen for proteolysis. Finally, only antioxidants targeting mitochondrial superoxide, rather than the commonly used antifibrinolytic drug TXA, could fully inhibit fibrinogen proteolysis and prevent coagulopathy.

Increased pro-inflammatory cytokines, leukocyte activation, and inflammation are common responses to both sepsis and trauma and have been reported to play an important role in associated coagulopathies [29,32]. In this study, to investigate mechanisms related to how leukocyte inflammation affected fibrinogen specifically, we chose a sterile inflammation LPS model both for its precise control of inflammatory response and lack of confounding aspects of tissue injury or active infection. Our intent was to provide a baseline view of the relationship between inflammation and fibrinogen using a foundational model of leukocyte inflammation that could be translated to more specific models of trauma and sepsis, where other effects of tissue injury, shock, and infection can be studied. Follow on studies of inflammation and fibrinogen behavior in response to polytrauma and hemorrhagic shock *in vivo* are currently underway and will be published separately.

Our findings support the existence of a “dual hit” process of altered fibrinogen form and function in response to leukocyte inflammation. First, oxidants generated by activated leukocytes directly oxidize fibrinogen Aα and Bβ chains. This oxidative stress may directly prime fibrinogen for proteolysis by oxidative modification and/or induce intracellular signaling events leading to expression of fibrinolytic proteases by leukocytes. Since antioxidants inhibiting mitochondrial superoxide inhibited both oxidation and proteolysis, it appears that superoxide is a key initiator of the first hit and that the second proteolytic hit is dependent upon its generation. “Two hit” models have been proposed for many different disease processes that include inflammation, including sepsis and trauma-induced multiorgan failure. After severe trauma as well as sepsis, the inflammatory response is “primed” so that any additional minor inflammatory stimulus triggers an exaggerated response [33]. Neutrophils have been implicated by their priming towards generation of oxidants. Our data suggest for the first time that a similar neutrophil-dependent oxidative priming process may contribute acutely to the development of TIC and the coagulopathy of sepsis where hypoperfusion and tissue injury act synergistically to produce coagulopathy [31].

It is curious that significant proteolytic breakdown of fibrinogen due to inflammation was delayed until >3 h after the onset of inflammation. This timing matches the time interval for which TXA has been found to be beneficial for reducing mortality in trauma patients [34]. This could be coincidence; however, our two-hit hypothesis of inflammatory coagulopathy does potentially explain this narrow therapeutic window as the delay required for significant leukocyte inflammatory gene activation and production of fibrinolytic enzymes leading to fibrinogen degradation. After 3 h, with significant degradation of fibrinogen already being present, the efficacy of antifibrinolytic drugs would logically be reduced. Antifibrinolytics combined with aggressive fibrinogen replacement would likely be required to restore coagulation function in this setting. Other work examining the effects of fibrinogen oxidation by NMR demonstrated *in vitro* that adding oxidized fibrinogen to plasma changed the behavior of the bulk solution, altered fibrin clot structure, and induced coagulopathy [35]. Linear positive relationships were found between concentration of oxidized fibrinogen and fibrin polymerization in plasma, leading to significantly altered fibrin polymerization at concentrations above 30–40%. Therefore, we do speculate that early and aggressive fibrinogen replacement would be beneficial to minimize the oxidized fraction of fibrinogen in plasma early during the oxidative phase. It follows logically that much more fibrinogen supplementation would be required later when oxidation is advanced and significant fibrinogen degradation is also present. We also found that TXA could not prevent proteolysis of the fibrinogen Aα-chain, which was associated with early prolongation of thrombin and reptilase times. This is not surprising, as TXA interacts at the Kringle 5 domain of plasminogen and only antifibrinolytics inhibiting the plasmin active site have been shown to prevent fibrinogen Aα-C domain cleavage [36,37].

These findings suggests that new approaches that include alternative antifibrinolytic drugs, antioxidants, and anti-inflammatory agents may be needed to fully preserve fibrinogen function during acute systemic inflammation. Studies antioxidant drug cocktails for inflammatory conditions have met with mixed results. Two observational studies reported remarkable efficacy with high-dose vitamin C, thiamine, and hydrocortisone therapy in critically ill septic patients [38,39]. However, follow-on randomized controlled trials found no benefit for these antioxidant and anti-inflammatory-based therapies [[38], [39], [40], [41], [42]]. Our results may provide new emphasis on the timing of such therapies. Our two-hit hypothesis suggests that to prevent irreversible changes, such therapies might be required very early after the onset of inflammation. While this may be more useful in trauma, where the time of injury is generally known, it may also be important in other inflammatory conditions such as sepsis, where minimizing time to fluid resuscitation and antibiotics are already known to be strongly associated with improved outcomes [43]. In addition, we found that NAC provided robust protection of both fibrinogen oxidation and degradation. This result is exciting because NAC is a commonly used drug in both oral and intravenous form that improves hemodynamics and oxygen delivery during hepatic failure and is commonly used antidote for treatment of acetaminophen toxicity in humans [44,45]. NAC is also generally well tolerated with minimal side effects. Therefore, NAC may also be a promising drug candidate for clinical repurposing for use to prevent coagulopathy during acute inflammation.

Regarding the specific source of oxidants generated during leukocyte inflammation, MPO has been reported to need NOX-derived superoxide to produce hypochlorous acid [46,47]. However, in our study, we found that MPO was activated and significant oxidation of fibrinogen took place after only 1 h, while NOX was activated only after 3 h. This time discrepancy supports that superoxide required for generation of hypochlorous acid by MPO was provided by mitochondria rather than NOX. This is important because our finding that mitochondrial superoxide combined with MPO-derived ROS was required to ultimately degrade and fragment fibrinogen.

We showed that fibrinogen Aα and Bβ are very susceptible and labile for oxidative modification and proteolysis and fibrinogen γ is very resistant to these two events by activated leukocytes. Their preference for oxidation and degradation may be due to the composition of amino acids of these fibrinogen isomers and their solvency. Fibrinogen α and β subunits are enriched with basic amino acids like histidine, lysine and arginine which are easily oxidized and target for many proteases while fibrinogen γ is composed of more acidic amino acids like aspartate and glutamate which are relatively more resistant to oxidation and proteolysis. Solvation may also contribute to the relative resistance to oxidation and proteolysis of the individual fibrinogen chains. Alpha-C domains are unordered, having increased flexibility due to a series of repeat sequences [48]. Thus, the flexibility of the alpha-C region increases solvation and promotes access by oxidants and proteolytic enzymes whereas other susceptible amino acids of the beta and gamma chains tend to be more buried within coil-coil regions.

Our study is limited in several ways. First, further work is required to determine what specific oxidative modifications are taking place on fibrinogen in response to leukocyte-induced inflammation. We have demonstrated increased methionine sulfoxide formation on the fibrinogen Aα-C domain in trauma patients with coagulopathy [17]. However, given the significant carbonyl formation noted in response to leukocyte inflammation, there are likely other oxidative modifications taking place that require identification. In addition, we found that intracellular gene signaling pathways involving NFκB *trans*-activation were activated in leukocytes that corresponded with timing of fibrinogen proteolysis. Partial efficacy of TXA to prevent fibrinogen Bβ-chain proteolysis suggests that plasmin was the proteolytic enzyme generated. However, further investigation is required to positively identify the culprit protease(s). Our results also support the need for additional clinical trials investigating potential benefit of early and aggressive fibrinogen replacement to prevent and treat coagulopathy in diseases associated with significant inflammation, including trauma and sepsis.

In conclusion, fibrinogen is sequentially oxidized and degraded during exposure to leukocyte activation and inflammation, both *in vivo* and *in vitro*. These events are governed by generation of mitochondrial superoxide and MPO-associated oxidants by leukocytes and can induce significant changes in fibrinogen coagulability and contribute to coagulopathy.

## Authorship

C.Y.H., T.J.P., X.W., K.M.R. and A.E.J. conducted the experiments. All authors interpreted the data and assisted with editing the manuscript. C.Y.H., S.A.S. and N.J.W. designed the experiments, supervised the work, interpreted the data, and wrote the manuscript.

## Declaration of competing interest

N.J.W. has received funding from U.S. NIH, DoD, and is shareholder in Stasys Medical Corp, and consultant to Velico Medical Corp. All other authors declare no conflict of interest.
