# Supplementary material for: Leukocyte activation primes fibrinogen for proteolysis by mitochondrial oxidative stress
Source: Redox Biol. 2022 Feb 10;51:102263. doi: 10.1016/j.redox.2022.102263 (PMC8844908; doi:10.1016/j.redox.2022.102263)
Supplement: Multimedia component 1 [file mmc1.docx]

**
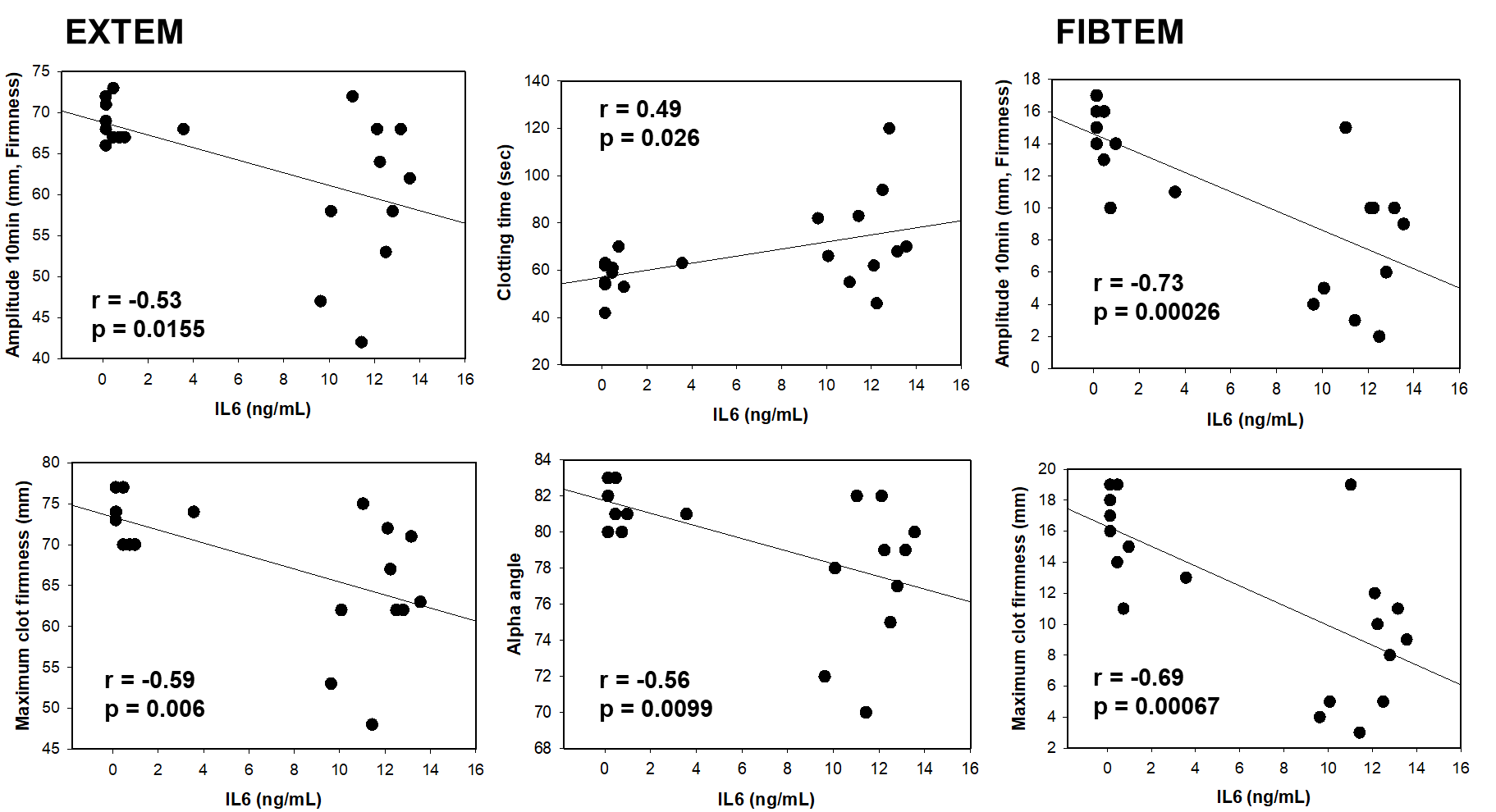
**

**Supplemental Figure I. Correlation between IL6 and firmness.** Whole blood and plasma were collected at the indicated time points from LPS-injected rats (n=5). Amplitude, clotting time, α-angle, and MCF were measured in FIBTEM and EXTEM. Relationships between IL6 and amplitude, clotting time, α-angle, or MCF, were determined by linear regression analysis using Pearson correlation coefficient.

**Supplemental Figure II. Validation of gating strategy.** Neutrophils, lymphocytes, and monocytes were identified from peripheral blood cells of normal healthy donors with the following high dimensional immunophenotyping; Neutrophils (CD45+, CD3/CD19−, CD16b+, CD14−), lymphocytes (CD45+, CD3/CD19+, CD16b−, CD14−) and monocytes (CD45+, CD3/CD19−, CD16b−, CD14−). Representative flow cytometry images from healthy donors (n=6) were shown.

**
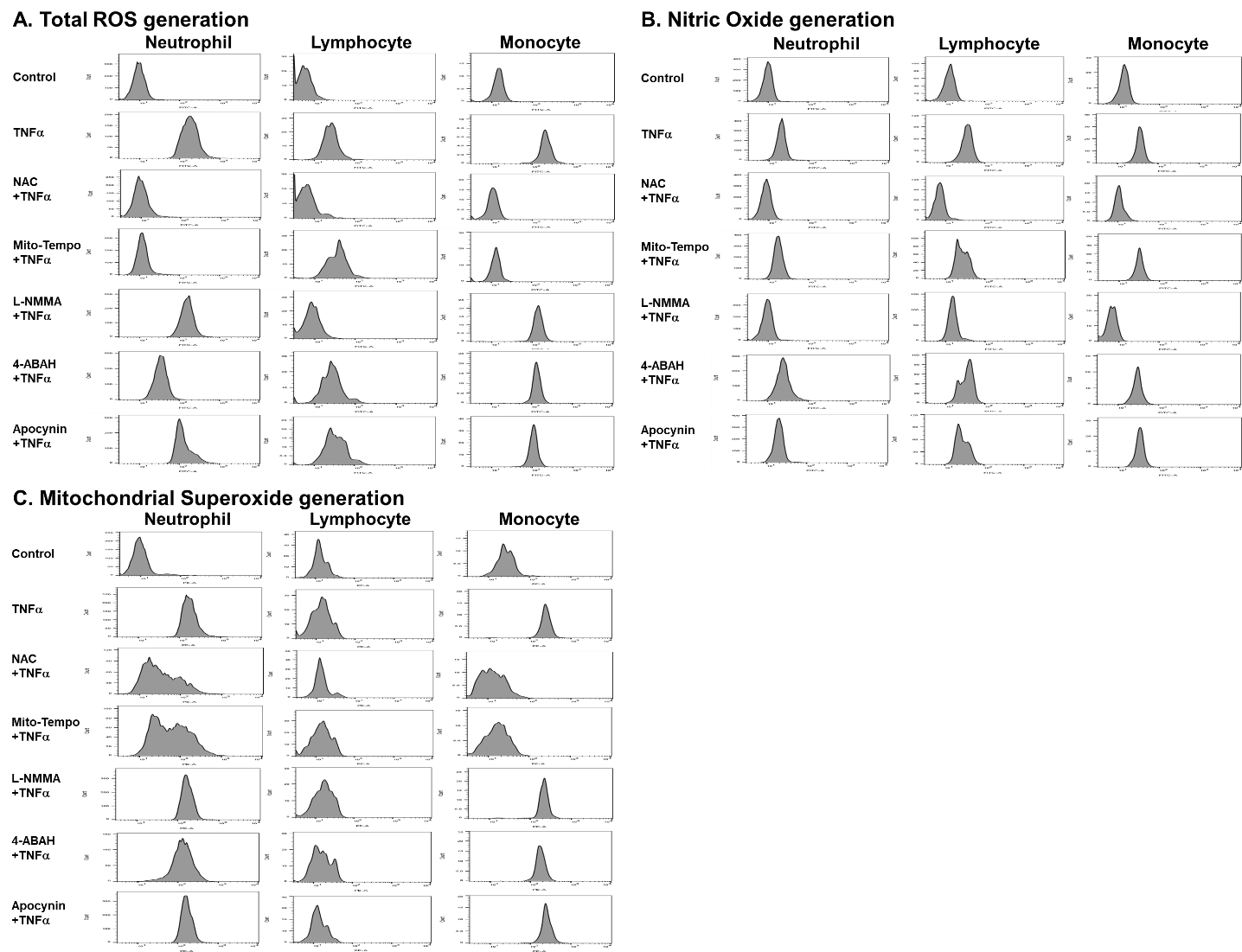
**

**Supplemental Figure III. Histograms of oxidative stress measured by fluorescence-based flow cytometry analysis in TNFα-activated leukocytes.** TNFα-activated leukocytes from healthy donors (n=6) were incubated with indicated various antioxidants (NAC; a pan antioxidant, Mito-Tempo; an antioxidant for mitochondrial superoxide, L-NMMA; an antioxidant for NOS, 4-ABAH; an antioxidant for MPO, apocynin; an antioxidant for NOX) for 3 hours. (*A-C*) Total ROS, NO and mitochondrial superoxide were measured as described in methods. Each panel are representative of 6 independent experiments.


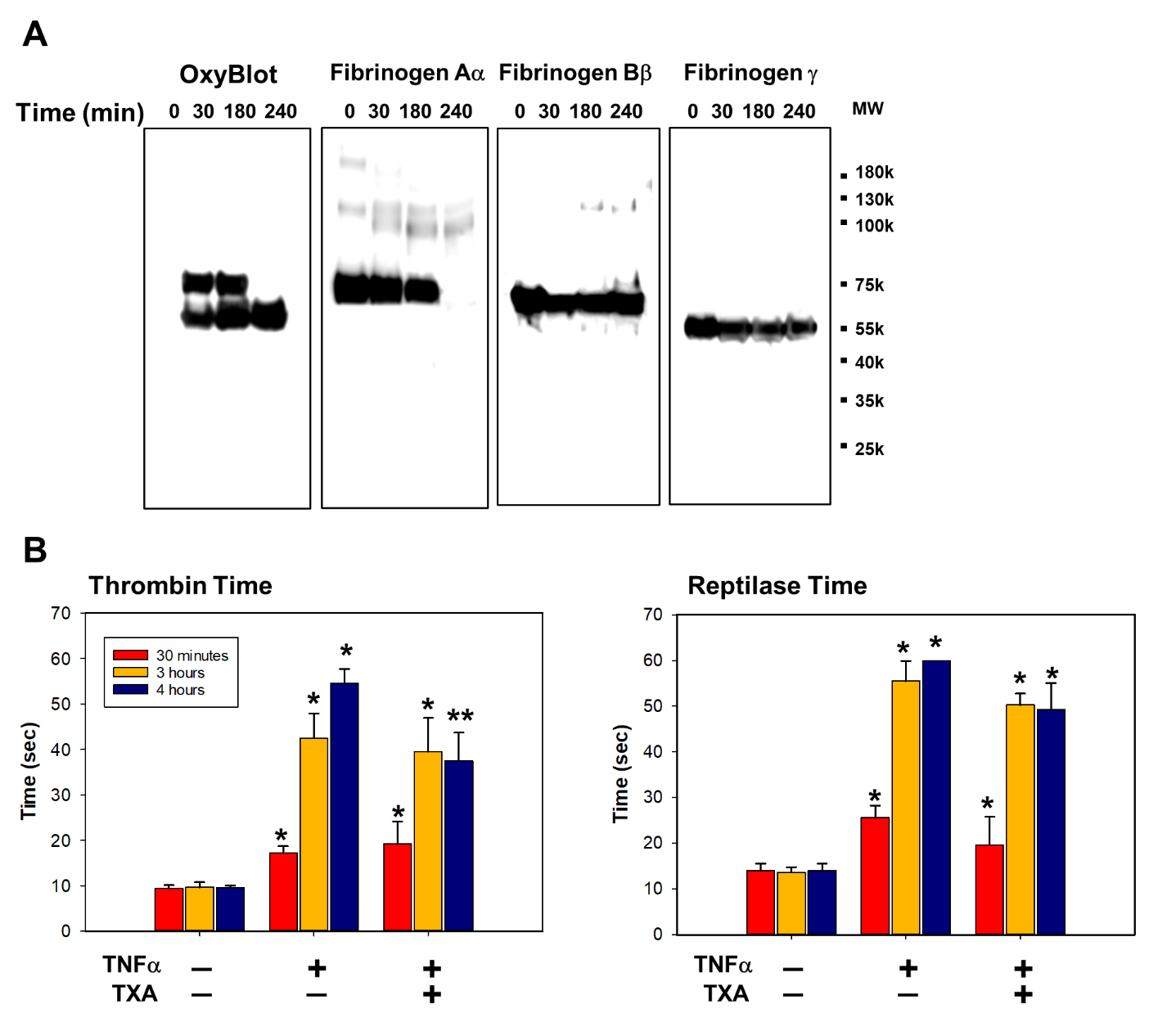


**Supplemental Figure IV. Tranexamic acid (TXA) does not protect oxidation of fibrinogen Aα and Bβ and only inhibits proteolysis of fibrinogen Bβ in TNFα- activated leukocytes.** TNFα-activated leukocytes from healthy donor (n=6) were incubated with fibrinogen and TXA for indicated time periods. (*A*) Conditioned media was analyzed by OxyBlot and immunoblotting using fibrinogen Aα, Bβ and γ – specific antibodies. Data images are representative of 3 independent experiments. (*B*) Conditioned media was analyzed for thrombin and reptilase time were measured as described in methods (n=6, mean ± SEM). *p < 0.001 vs. control. **p < 0.001 vs. TNFα. ANOVA and Bonferroni post-hoc test.
